# Membrane Lipids in the Thyroid Comparing to Those in Non-Endocrine Tissues Are Less Sensitive to Pro-Oxidative Effects of Fenton Reaction Substrates

**DOI:** 10.3389/fmolb.2022.901062

**Published:** 2022-06-03

**Authors:** Jan Stępniak, Aleksandra Rynkowska, Małgorzata Karbownik-Lewińska

**Affiliations:** ^1^ Department of Oncological Endocrinology, Medical University of Lodz, Lodz, Poland; ^2^ Polish Mother’s Memorial Hospital—Research Institute, Lodz, Poland

**Keywords:** Fenton reaction, lipid peroxidation, oxidative damage, thyroid, melatonin

## Abstract

Iron is an essential microelement for the proper functioning of many organs, among others it is required for thyroid hormone synthesis. However, its overload contributes to the increased formation of reactive oxygen species via Fenton chemistry (Fe^2+^+H_2_O_2_→Fe^3+^+^˙^OH + OH^−^), and it is potentially toxic. Individual organs/tissues are affected differently by excess iron. The excessive absorption of iron with subsequent deposition in various organs is associated with diseases such as hemochromatosis. Such an iron deposition also occurs in the thyroid gland where it can disturb thyroid hormone synthesis. In turn, melatonin is an effective antioxidant, which protects against oxidative damage. This study aims to check if lipid peroxidation resulting from oxidative damage to membrane lipids, is caused by Fenton reaction substrates, and if protective effects of melatonin differ between the thyroid and various non-endocrine porcine tissues (liver, kidney, brain cortex, spleen, and small intestine). To mimic the conditions of iron overload, Fe^2+^ was used in extremely high concentrations. Homogenates of individual tissues were incubated together with Fenton reaction substrates, i.e., FeSO_4_ (9.375, 18.75, 37.5, 75, 150, 300, 600, 1,200, 1,800, 2,100, 2,400, 3,000, 3,600, 4,200, and 4,800 µM)+H_2_O_2_ (5 mM), either without or with melatonin (5 mM). The concentration of malondialdehyde+4-hydroxyalkenals (MDA+4-HDA), as the LPO index, was evaluated by a spectrophotometrical method. Fenton reaction substrates increased concentrations of LPO products in all chosen tissues. However, in the thyroid, compared to non-endocrine tissues, the damaging effect was generally weaker, it was not observed for the two lowest concentrations of iron, and the LPO peak occurred with higher concentrations of iron. Melatonin reduced experimentally induced LPO in all examined tissues (without differences between them), and these protective effects did not depend on iron concentration. In conclusion, membrane lipids in the thyroid compared to those in non-endocrine tissues are less sensitive to pro-oxidative effects of Fenton reaction substrates, without differences regarding protective effects of melatonin.

## Introduction

Iron is a critical micronutrient in mammalian organisms, and it is a cofactor for many biological reactions. As a redox-active transition metal, it acts as an electron donor and acceptor in a plethora of fundamental cellular processes, such as oxygen transport, DNA and RNA synthesis, cell proliferation, and energy metabolism. In mammals, all iron is obtained from digestion of food where it exists largely in two forms, i.e., non-heme iron in the relatively non-toxic ferric form (Fe^3+^), derived mainly from plant-based foods and animal products, and heme iron in a more reactive and toxic form of ferrous ion (Fe^2+^), derived from the breakdown of hemoglobin and myoglobin in animal tissues. Despite the fact that non-heme iron is usually much less well absorbed than heme iron, it constitutes most of the human dietary iron, even in meat-eating populations (Hurrell and Egli, 2010; Abbaspour et al., 2014).

The adult well-nourished human body contains approx. 3–5 g of iron. Under healthy conditions, most of it is bound to some form of a ligand. It is estimated that up to 80% of body iron is present in red blood cell hemoglobin, while another approximately 20% is stored in the form of ferritin and heme within hepatocytes and macrophages of the liver and spleen (Hentze et al., 2004). The remaining small amount (<1%) of the iron in the human body is found in various proteins such as cytochromes (Lane et al., 2015).

This iron compartmentalization is crucial for organism homeostasis since “free” (catalytically active) iron can react with hydrogen peroxide (H_2_O_2_) via the Fenton chemistry (Fe^2+^ + H_2_O_2_ → Fe^3+^ + ^˙^OH + OH^−^), the reaction directly related to the oxidative stress. The hydroxyl radical ( ^˙^OH) produced during this process is one of the most powerful oxidizing agents and can react—at a diffusion-controlled rate—with practically all subcellular components in the organism (Koppenol and Hider, 2019). The hydroxyl radical can react and consequently damage all biological macromolecules (lipids, proteins, nucleic acids, and carbohydrates) leading to cell dysfunction and death. It has been observed that the harmful effects of ^˙^OH may contribute to pathogenesis of cancer, atherosclerosis, or neurodegenerative diseases (Lipinski, 2011).

Improper iron compartmentalization and subsequent elevated levels of oxidative stress are often the results of accumulation and overload of this element. The human organism does not have any specific mechanism to remove excess iron. Iron elimination occurs only via non-regulated ways such as cell desquamation or bleeding; hence, its concentration is controlled only at the level of iron absorption. A number of diseases and pathological factors can lead to iron overload. The most common causes of iron overload are overconsumption of iron when it is in excess in the environment (Aranda et al., 2016), congenital disturbances of iron metabolism (hemochromatosis) (Girelli et al., 2021), or secondary hemochromatosis resulting from repeated blood transfusions in patients with beta-thalassemia (Ali et al., 2021) or with sickle cell anemia (Badawy et al., 2016). The growing literature demonstrates that also chronic hepatitis C may cause iron overload (Zou and Sun, 2017).

Iron overload and enhanced oxidative stress can lead to adverse effects in all tissues; however, it can be, especially severe in the thyroid when we take into consideration the “oxidative nature” of this gland. Hydrogen peroxide (one of the Fenton reaction substrates) is indispensable for biosynthesis of thyroid hormones, in which it serves as an electron acceptor at each stage of this process (Song et al., 2007). Therefore, H_2_O_2_ is generated in the thyroid in high concentrations which, in turn, can create favorable conditions for the Fenton reaction to occur.

Until now, Fenton reaction substrates were used very commonly to experimentally induce oxidative damage to macromolecules in different tissues (e.g., ovary, thyroid, and skin) with very high Fe^2+^ concentrations (Rynkowska et al., 2020, 2021), equal to these used in the present study.

This study aims to check if lipid peroxidation (LPO) resulting from oxidative damage to membrane lipids caused by Fenton reaction substrates and protective effects of melatonin differs between the thyroid and various non-endocrine porcine tissues (liver, kidney, brain cortex, spleen, and small intestine). While it is currently known that certain hormones can be synthesized by and secreted from nontraditional endocrine organs, still specific organs classified as endocrine glands are separated. They are traditionally defined as ductless glandular structures that release their hormonal secretions into the extracellular space, from where they can eventually enter the bloodstream; examples of classic endocrine glands are the thyroid gland, the pituitary gland, the adrenal gland, and the ovary, etc. (Hsiao et al., 2017; Holt et al., 2021). Regarding tissues, such as liver, kidney, brain cortex, spleen, and small intestine, which are used in the present study, do not fulfill the criteria of an endocrine gland; therefore, they can be called non-endocrine tissues. In fact, experimental and clinical studies to describe various processes occurring in endocrine versus non-endocrine tissues have been published before ( McCarthy et al., 2013; Xie et al., 2018). In the present study, we have chosen these non-endocrine tissues, which play important roles in iron absorption (intestine) and accumulation (liver and spleen) or iron overload. Iron overload plays a crucial role in the pathogenesis of diseases developing in the tissues, such as brain cortex (Belaidi and Bush, 2016) or kidney (Nakanishi et al., 2019). To simulate conditions of iron overload, Fe^2+^ ion was used in the present study in extremely high concentrations.

## Materials and Methods

### Ethical Considerations

In accordance with the Polish Act on the Protection of Animals Used for Scientific or Educational Purposes from 15 January, 2015 (which implements Directive 2010/63/EU of the European Parliament and the Council of 22 September 2010 on the protection of animals used for scientific purposes)—the use of animals to collect organs or tissues does not require the approval of the Local Ethics Committee. These animals are only subjected to registration by the center in which the organs or tissues were taken. Additionally, we did not use experimental animals; instead, porcine tissues were collected from animals at a slaughterhouse during the routine process of slaughter carried out for consumption.

### Chemicals

All chemicals applied in the study are of analytical grade and come from the following commercial sources: melatonin, ferrous sulfate (FeSO_4_), and hydrogen peroxide (H_2_O_2_)—Sigma (St. Louis, MO, United States); the ALDetect Lipid Peroxidation Assay Kit–Enzo Life Sciences, Inc. (Zandhoven, Belgium).

### Tissue Collection

Porcine tissues were collected from pigs slaughtered at the local slaughterhouse. Animals were treated according to the European Community Council Regulation (CE1099/2009) concerning protection of animals at the time of killing. All animals were sexually mature as determined by age (8–9 months) and body mass [118 ± 3.8 (SD) kg]. They were in good body condition and considered free of pathologies by the veterinary medical officer responsible for the health of animals and hygiene of the slaughterhouse. Immediately (in less than 5 min) after the slaughter, the tissues, i.e., thyroid, spleen, liver (from left lateral lobe), brain cortex, small intestine (jejunum and ileum), and kidney (renal cortex), were collected, frozen on solid CO_2_, and stored at −80°C till experimental procedure. Each experiment was repeated three times.

### Incubation of Tissue Homogenates

We homogenized individual tissues (thyroid, spleen, liver, brain cortex, small intestine, and kidney) in ice-cold 20 mM Tris–HCl buffer (pH 7.4) (10%, w/v), and then tissues were incubated (37°C, 30 min) in the presence of FeSO_4_ (9.375, 18.75, 37.5, 75, 150, 300, 600, 1,200, 1,800, 2,100, 2,400, 3,000, 3,600, 4,200, and 4,800 µM)+H_2_O_2_ (5 mM) without melatonin or with the addition of melatonin in its highest achievable *in vitro* concentration (due to limited solubility), i.e., 5 mM. After incubation, the samples were cooled on ice to stop the reaction.

### Assay of Lipid Peroxidation

The ALDetect Lipid Peroxidation Assay Kit was used to measure concentrations of malondialdehyde + 4-hydroxyalkenals (MDA+4-HDA), being the index of lipid peroxidation. To obtain supernatants, samples of homogenates were centrifuged (5,000×*g*, 10 min, 4°C). Next, 200 μL of supernatant was mixed with methanol: acetonitrile (1:3, v/v) solution (650 μL), containing N-methyl-2-phenylindole as a chromogenic reagent, and after that the sample was vortexed. In the next step, methanesulfonic acid (150 μL, 15.4 M) was added, and then incubation was conducted again (45°C, 40 min). The product of the reaction between MDA+4-HDA and N-methyl-2-phenylindole was a chromophore, which was measured by the spectrophotometrical method (at an absorbance of 586 nm) with the use of a 4-hydroxynonenal solution (10 mM) as the standard. The amount of protein was measured with the use of Bradford’s method (the standard was bovine albumin) (Bradford, 1976). We expressed the level of lipid peroxidation as the concentration of MDA+4-HDA (nmol)/mg protein.

### Statistical Analyses

We used the following statistical tests: one-way analysis of variance (ANOVA), followed by the Student–Neuman–Keuls test, or an unpaired *t*-test. *p* < 0.05 was accepted as the level of statistical significance. We presented the results as means ± SE.

## Results

**FIGURE 1:**
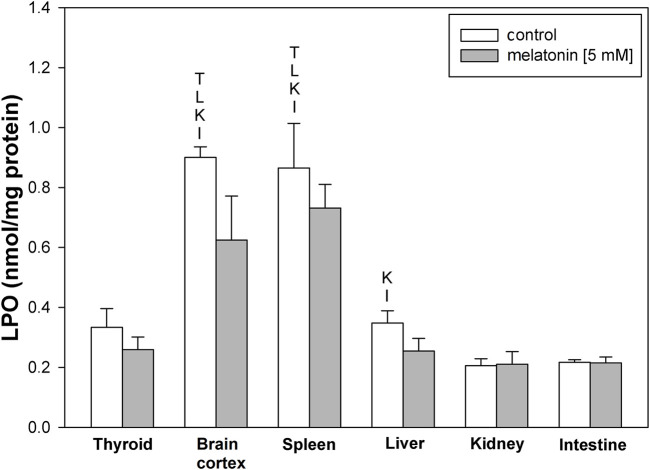
Concentrations of lipid peroxidation products (MDA+4-HDA) in the homogenates of porcine tissues (thyroid, brain cortex, spleen, liver, kidney, and intestine) incubated without any substance (control; white bars) or with melatonin (5 mM) (gray bars). T–*p* < 0.05 vs. respective control in the thyroid; L–*p* < 0.05 vs. respective control in the liver; K–*p* < 0.05 vs. respective control in the kidney; I–*p* < 0.05 vs. respective control in the intestine. Differences in LPO levels between control and melatonin are not statistically significant in particular tissues. Statistical differences between particular tissues after melatonin exposure are not marked.

LPO levels under basal conditions were higher in the brain cortex and spleen than in other examined tissues (Figure 1). Incubation with melatonin did not change basal levels of LPO significantly in any of the examined tissues (Figure 1).

**FIGURE 2:**
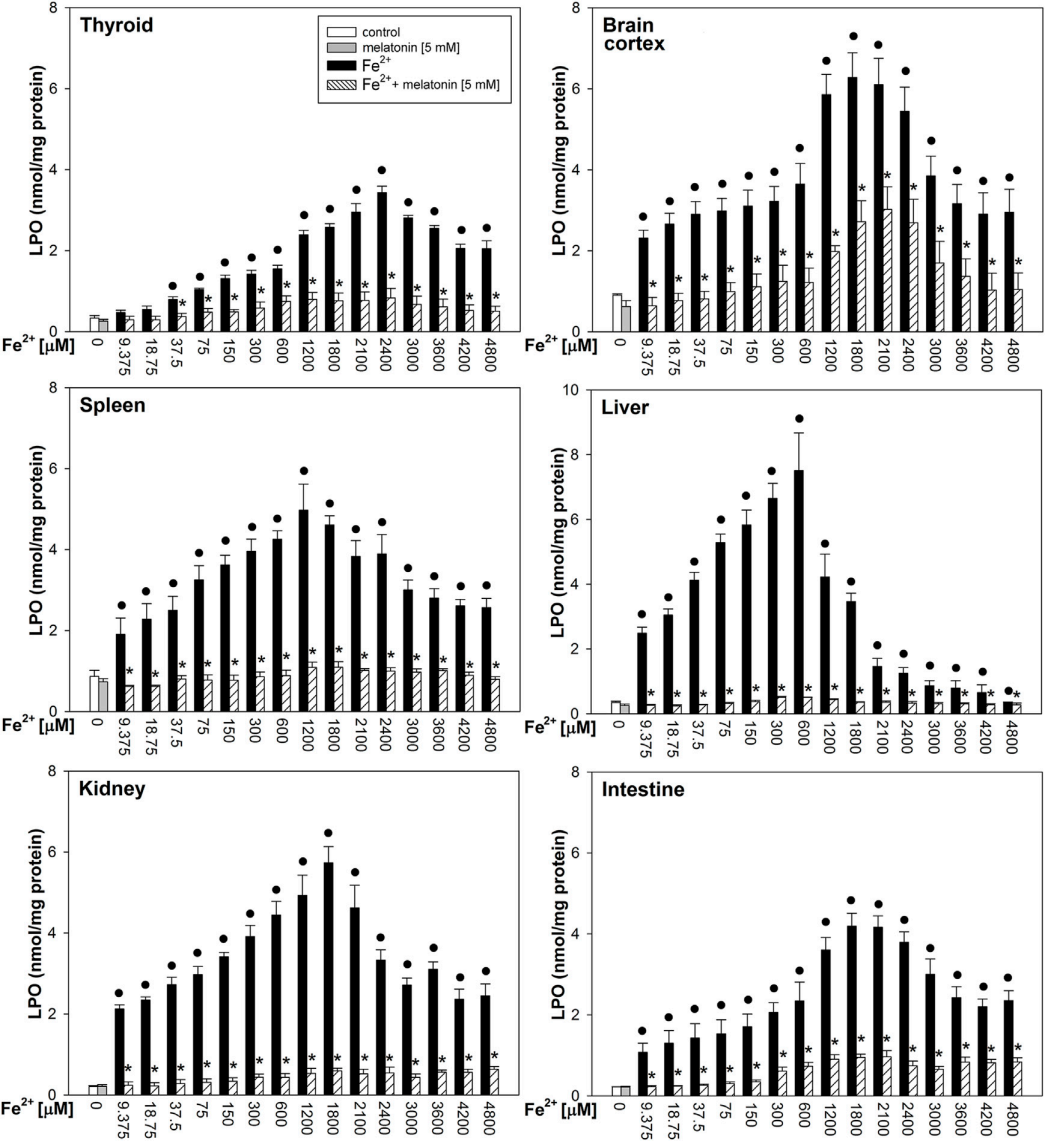
Concentrations of lipid peroxidation products (MDA+4-HDA) in the homogenates of porcine tissues (thyroid, brain cortex, spleen, liver, kidney, and intestine), incubated without any substance (control; white bars) or with melatonin (5 mM) (gray bars) or with Fe^2+^ (9.375, 18.75, 37.5, 75, 150, 300, 600, 1,200, 1,800, 2,100, 2,400, 3,000, 3,600, 4,200, and 4,800 µM) + H_2_O_2_ (5 mM) (black bars) or with Fe^2+^ (9.375, 18.75, 37.5, 75, 150, 300, 600, 1,200, 1,800, 2,100, 2,400, 3,000, 3,600, 4,200, and 4,800 µM) + H_2_O_2_ (5 mM) with melatonin (5 mM) (striped bars). *p* < 0.05 vs. respective control (without any substance); *p < 0.05 vs. Fe^2+^ in the same concentration. Statistical differences between Fe^2+^+H_2_O_2_+melatonin (stripped bars) vs. melatonin (gray bars) in particular tissues are not marked.

**FIGURE 3:**
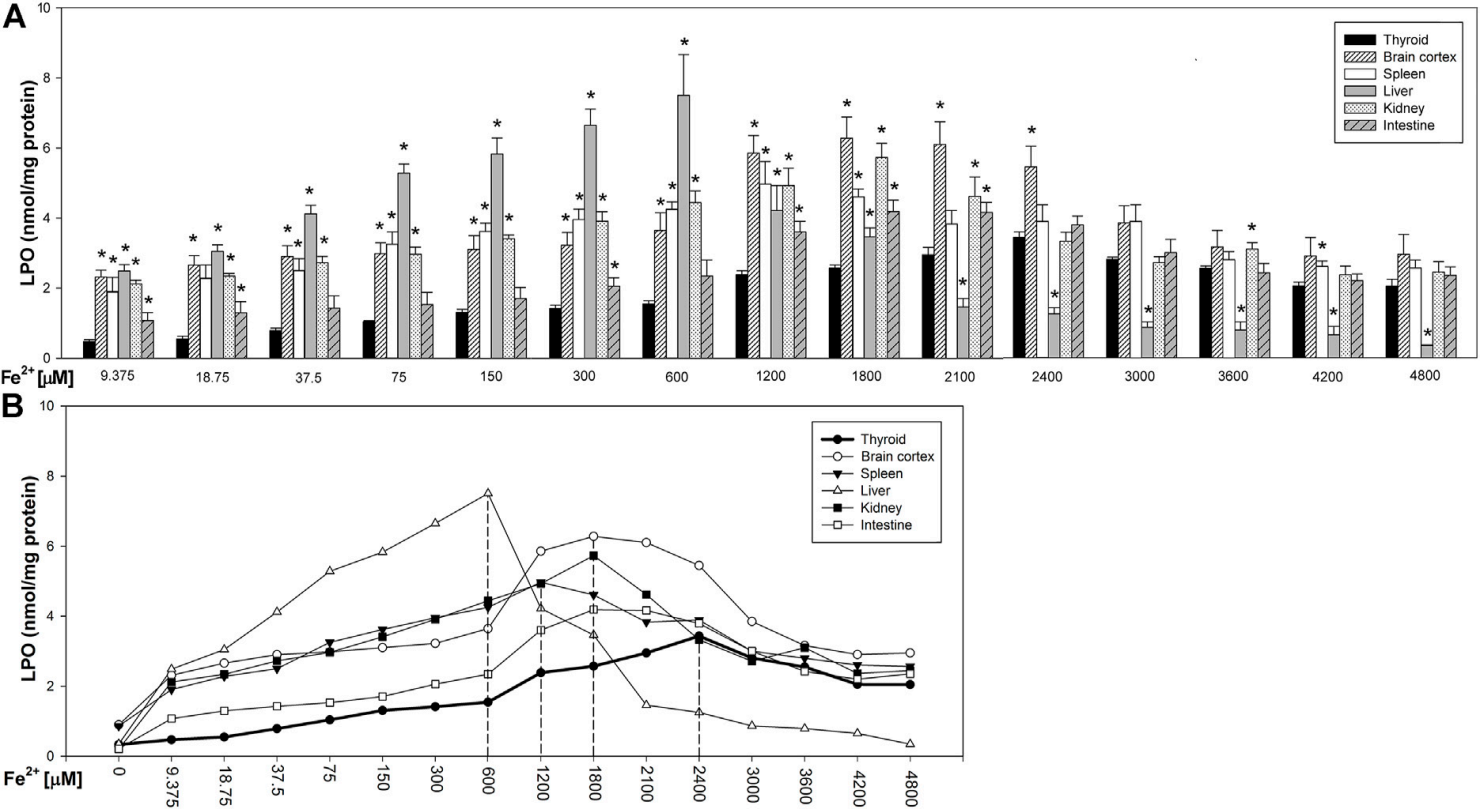
**(A)** Concentrations of lipid peroxidation products (MDA+4-HDA) in the homogenates of porcine tissues (thyroid, brain cortex, spleen, liver, kidney, and intestine), incubated with Fe^2+^ (9.375, 18.75, 37.5, 75, 150, 300, 600, 1,200, 1,800, 2,100, 2,400, 3,000, 3,600, 4,200, and 4,800 µM) + H_2_O_2_ (5 mM). *p < 0.05 vs. respective concentration in the thyroid. **(B)** Line graph representing concentrations of lipid peroxidation products (MDA+4-HDA) in the homogenates of porcine tissues (thyroid, brain cortex, spleen, liver, kidney, and intestine), incubated with Fe^2+^ (9.375, 18.75, 37.5, 75, 150, 300, 600, 1,200, 1,800, 2,100, 2,400, 3,000, 3,600, 4,200, and 4,800 µM) + H_2_O_2_ (5 mM). Vertical dashed lines indicate that iron concentration at which the LPO peak occurs for each individual tissue. Data points represent mean values of independent experiments analogically to results presented in Figure 3.

Fenton reaction substrates increased LPO levels in all examined tissues and—except for the thyroid—in all used concentrations of Fe^2+^ (Figure 2). Namely, in the thyroid, the damaging effect was not observed for the two lowest concentrations of iron (9.375 and 18.75 µM) (Figure 2). Additionally, compared to that in non-endocrine tissues, Fenton reaction-induced damage in the thyroid was weak for the increasing Fe^2+^ concentrations up to 1,800 μM; although with the increasing Fe^2+^ concentrations above 1,800 µM, these differences gradually disappeared (Figure 3A). Importantly, the LPO peak occurred “later,” i.e., with higher concentrations of iron, whereas the LPO peak was observed in the thyroid at an Fe^2+^ concentration of 2,400 μM. In the liver, the LPO peak was recorded at an Fe^2+^ concentration of 600 µM (four times lower than that in the thyroid), in the spleen—at an Fe^2+^ concentration of 1,200 µM (two times lower than that in the thyroid), and in the kidney, brain cortex, and intestine—at an Fe^2+^ concentration of 1,800 µM (lower by 25% than that in the thyroid) (Figures 3A,B).

Melatonin reduced LPO levels induced by Fenton reaction substrates in all tissues; these protective effects did not significantly differ between tissues, and the protective effects of melatonin did not depend on iron concentration (Figure 2).

## Discussion

In our earlier studies, we have observed that the thyroid gland is less sensitive to the damaging effects of iron than the ovary which is also an endocrine gland (Rynkowska et al., 2020). We have also evaluated the damaging effect of KIO_3_ and have found that KIO_3_-induced LPO is significantly lower in the thyroidal tissue than non-endocrine tissues and the ovary (Iwan et al., 2021a). Our present results confirm the results cited previously and clearly indicate that membrane lipids in the thyroid are less sensitive to pro-oxidative events occurring in this gland. Such findings suggest that the thyroid gland has developed more effective (than other tissues) protective mechanisms for maintaining redox homeostasis.

The thyroid gland constitutes a specific organ in such a sense that oxidative reactions are absolutely required for thyroid hormone synthesis. Hydrogen peroxide is generated for the needs of thyroid hormone biosynthesis by NADPH oxidases, especially by dual oxidase 2 (DUOX2) (Pachucki et al., 2004), and it is synthesized in a higher amount than this which is required for proper iodide incorporation into thyroid hormones. This apparent discrepancy may be associated with the relatively high Michaelis–Menten constant of thyroperoxidase (TPO, a clue enzyme in thyroid hormone synthesis) for H_2_O_2_, resulting in relatively higher concentrations of H_2_O_2_ (as a substrate) indispensable for proper activation of TPO (Song et al., 2007). Interestingly, in our experimental study on sexual dimorphism, we have found that thyroid follicular cells from female thyroids are exposed to higher H_2_O_2_ concentrations than male thyroids, which probably results from the higher activity of NOX/DUOX enzymes in the female thyroid (Stepniak et al., 2018), and it is in agreement with the assumption that higher prevalence of thyroid diseases in women is associated with stronger oxidative stress. H_2_O_2_ is primarily a toxic compound with a relatively long half-life which, as a non-polar molecule, is able to diffuse across biological membranes. Because it is an oxidizing agent, it is in power to induce damage to biological macromolecules such as DNA, lipids, and proteins, consequently leading to mutagenesis and apoptosis (Song et al., 2007). Therefore, to ensure the proper functioning of the thyroid, sources of H_2_O_2_ and other potentially pro-oxidative compounds need to be under strict control. Therefore, some adaptive mechanisms have developed in the thyroid, such as specific localization of potentially dangerous processes (e.g., production of H_2_O_2_) and compartmentalization of potentially damaging elements (Szanto et al., 2019), as well as formation of antioxidants and ROS-scavenging systems. Regarding the last mentioned adaptive mechanism, responsible for this are mainly redox-controlling enzymes such as peroxiredoxins, glutathione peroxidases, and catalase. As catalase is rather weakly expressed in the thyroid, H_2_O_2_ degradation is carried out in this organ mainly by the enzymes from the glutathione peroxidases family (Song et al., 2007; Schweizer et al., 2008). This enzymatic antioxidative system is additionally supported by the contribution of molecules such as ascorbic acid, polyphenolic compounds, coenzyme Q10, β-carotene, retinol, and tocopherol (Kochman et al., 2021).

Under basal conditions, these thyroid systems effectively fulfill their antioxidant role. In our both studies (the present and the previous by Iwan et al., 2021a), we have observed that the basal level of LPO in the thyroid gland is not higher than that in other tissues. However, under any pathological condition associated either with endogenous abnormalities or exposure to exogenous pro-oxidants, this redox balance may be disrupted and, in consequence, the level of oxidative damage can be increased, resulting in various diseases (Valko et al., 2007), such as cancer (Ziech et al., 2011). A phenomenon that can definitely lead to disruption of redox homeostasis in the thyroid is iron overload. Consistently, we have shown earlier that Fe^2+^, than H_2_O_2_, damages more strongly both nuclear DNA and membrane lipids (Stepniak et al., 2013). Hepatic iron concentration in patients with asymptomatic and symptomatic hemochromatosis is approximately 8 to 15 times higher than that in healthy subjects—36–550 μmol/g (36–550 mM) in dry weight tissue (Bacon et al., 2011). Whereas in healthy individuals blood iron concentration is below 150 μg/dl, it is 150–300 μg/dl (0.026–0.053 mM) in subjects with hemochromatosis (Bacon et al., 2011). To mimic conditions of iron overload, in the present study, we have used the range of iron concentrations from 0.009 to 4.8 mM, which corresponds to the aforementioned.

In case of disrupted redox homeostasis, different exogenous antioxidants can be considered to be used. One of the most famous antioxidants is melatonin (N-acetyl-5-methoxytryptamine), which is produced in the organism mostly by the pineal gland, but it is also available as an exogenous substance. This molecule is confirmed to be a very effective antioxidant and free radical scavenger which prevents oxidative damage not only in the thyroid (Lewinski and Karbownik, 2002; Karbownik et al., 2005; Kokoszko-Bilska et al., 2014; Zasada and Karbownik-Lewinska, 2015; Iwan et al., 2021a, 2021b; Stepniak et al., 2021) but also in many other tissues and organs (Karbownik et al., 2000, 2001a, 2001b, 2006; Gitto et al., 2001; Osuna et al., 2002; Mogulkoc et al., 2006; Reiter et al., 2017). In the current study, we have observed that melatonin reduced LPO induced by Fenton reaction substrates in all examined tissues, and this protective effect was independent of iron concentration. These results confirm a well-known fact that melatonin is very effective in protecting against even these effects of iron which are caused by its extremely high concentrations (corresponding to those iron concentrations found in patients with hemochromatosis). It should be underlined that in our study melatonin did not reduce LPO levels below the physiological threshold in control groups of any examined tissue. This additionally supports the statement that this indoleamine is a distinctive antioxidant, which does not affect physiological processes, whereas it is effective under conditions with additional oxidative abuse.

Our study is probably the first attempt to compare the damaging effects of high iron concentrations in the thyroid and in various non-endocrine tissues under *in vitro* conditions. It should be stressed that the lower sensitivity of thyroidal membrane lipids, compared to that of membrane lipids in non-endocrine tissues, in response to high iron concentrations, is similar to previously observed pro-oxidative effects of KIO_3_ (Iwan et al., 2021a). Therefore, we suppose that membrane lipids (and possibly other biological macromolecules) in the thyroid reveal higher resistance to any external pro-oxidative agent than those in other tissues. These differences in sensitivities of given tissues to pro-oxidative factors result presumably from oxidative/antioxidative processes which normally (under physiological conditions) occur in the tissue. Because oxidative processes occur in the thyroid gland at a high level (Karbownik-Lewińska and Kokoszko-Bilska, 2012), this endocrine gland has developed an adaptive mechanism and, therefore, it is probably better prepared to protect against damaging effects of pro-oxidants.

This study has some limitations. First, our study was conducted using tissue homogenates, so our results may not be directly extrapolated under *in vivo* conditions, especially into the human organism. However, some directions of action of a given agent observed *in vitro* should be taken into account also under *in vivo* conditions. Second, we used only one experimental method to measure oxidative damage to membrane lipids, i.e., a spectrophotometric assay, evaluating lipid peroxidation by measuring MDA + 4-HDA. Although this experimental method has some disadvantages, it is very reliable and is commonly used in studies on oxidative stress.

## Data Availability

The raw data supporting the conclusion of this article will be made available by the authors, without undue reservation.

## References

[B1] Abbaspour N. Hurrell R. Kelishadi R. (2014). Review on Iron and its Importance for Human Health. J. Res. Med. Sci. 19, 164–174. 24778671 PMC3999603

[B2] Ali S. Mumtaz S. Shakir H. A. Khan M. Tahir H. M. Mumtaz S. (2021). Current Status of Beta-Thalassemia and its Treatment Strategies. Mol. Genet. Genomic. Med. 9, e1788. 10.1002/mgg3.1788 34738740 PMC8683628

[B3] Aranda N. Fernandez-Cao J. C. Tous M. Arija V. (2016). Increased Iron Levels and Lipid Peroxidation in a Mediterranean Population of Spain. Eur. J. Clin. Invest. 46, 520–526. 10.1111/eci.12625 26999720

[B4] Bacon B. R. Adams P. C. Kowdley K. V. Powell L. W. Tavill A. S. (2011). Diagnosis and Management of Hemochromatosis: 2011 Practice Guideline by the American Association for the Study of Liver Diseases. Hepatology 54, 328–343. 10.1002/hep.24330 21452290 PMC3149125

[B5] Badawy S. M. Liem R. I. Rigsby C. K. Labotka R. J. DeFreitas R. A. Thompson A. A. (2016). Assessing Cardiac and Liver Iron Overload in Chronically Transfused Patients with Sickle Cell Disease. Br. J. Haematol. 175, 705–713. 10.1111/bjh.14277 27507431

[B6] Belaidi A. A. Bush A. I. (2016). Iron Neurochemistry in Alzheimer's Disease and Parkinson's Disease: Targets for Therapeutics. J. Neurochem. 139, 179–197. 10.1111/jnc.13425 26545340

[B7] Bradford M. M. (1976). A Rapid and Sensitive Method for the Quantitation of Microgram Quantities of Protein Utilizing the Principle of Protein-Dye Binding. Anal. Biochem. 72, 248–254. 10.1016/0003-2697(76)90527-3 942051

[B8] GirelliD.BustiF.BrissotP.CabantchikI.MuckenthalerM. U.PortoG. (2021). Hemochromatosis Classification: Update and Recommendations by the BIOIRON Society. Blood 139, 3018–3029. 10.1182/blood.2021011338 34601591

[B9] Gitto E. Tan D. X. Reiter R. J. Karbownik M. Manchester L. C. Cuzzocrea S. (2001). Individual and Synergistic Antioxidative Actions of Melatonin: Studies with Vitamin E, Vitamin C, Glutathione and Desferrioxamine (Desferoxamine) in Rat Liver Homogenates. J. Pharm. Pharmacol. 53, 1393–1401. 10.1211/0022357011777747 11697548

[B10] Hentze M. W. Muckenthaler M. U. Andrews N. C. (2004). Balancing Acts. Cell. 117, 285–297. 10.1016/s0092-8674(04)00343-5 15109490

[B11] Holt R. I. G. Hanley N. A. (2021). Overview of Endocrinology” in Essential Endocrinology and Diabetes (Essentials). Hoboken, NJ: John Wiley & Sons, 1–39.

[B12] Hsiao E. C. Gardner D. G. (2017). Hormones and Hormone Action” in Greenspan's Basic and Clinical Endocrinology. New York, NY: McGraw-Hill Education, 1–28.

[B13] Hurrell R. Egli I. (2010). Iron Bioavailability and Dietary Reference Values. Am. J. Clin. Nutr. 91, 1461S–1467S. 10.3945/ajcn.2010.28674f 20200263

[B14] Iwan P. Stepniak J. Karbownik-Lewinska M. (2021b). Melatonin Reduces High Levels of Lipid Peroxidation Induced by Potassium Iodate in Porcine Thyroid. Int. J. Vitam. Nutr. Res. 91, 271–277. 10.1024/0300-9831/a000628 31842692

[B15] Iwan P. Stepniak J. Karbownik-Lewinska M. (2021a). Pro-Oxidative Effect of KIO3 and Protective Effect of Melatonin in the Thyroid-Comparison to Other Tissues. Life 11, 592. 10.3390/life11060592 34205777 PMC8234753

[B16] Karbownik M. Gitto E. Lewiñski A. Reiter R. J. (2001a). Relative Efficacies of Indole Antioxidants in Reducing Autoxidation and Iron-Induced Lipid Peroxidation in Hamster Testes. J. Cell. Biochem. 81, 693–699. 10.1002/jcb.1100 11329624

[B17] Karbownik M. Reiter R. J. Cabrera J. Garcia J. J. (2001b). Comparison of the Protective Effect of Melatonin with Other Antioxidants in the Hamster Kidney Model of Estradiol-Induced DNA Damage. Mutat. Research/Fundamental Mol. Mech. Mutagen. 474, 87–92. 10.1016/s0027-5107(00)00164-0 11239965

[B18] Karbownik M. Reiter R. J. Garcia J. J. Tan D. X. Qi W. Manchester L. C. (2000). Melatonin Reduces Rat Hepatic Macromolecular Damage Due to Oxidative Stress Caused by δ-aminolevulinic Acid. Biochimica Biophysica Acta (BBA) - General Subj. 1523, 140–146. 10.1016/s0304-4165(00)00110-0 11042377

[B19] Karbownik M. Stasiak M. Zasada K. Zygmunt A. Lewinski A. (2005). Comparison of Potential Protective Effects of Melatonin, Indole-3-Propionic Acid, and Propylthiouracil against Lipid Peroxidation Caused by Potassium Bromate in the Thyroid Gland. J. Cell. Biochem. 95, 131–138. 10.1002/jcb.20404 15723291

[B20] Karbownik M. Stasiak M. Zygmunt A. Zasada K. Lewiński A. (2006). Protective Effects of Melatonin and Indole-3-Propionic Acid against Lipid Peroxidation, Caused by Potassium Bromate in the Rat Kidney. Cell. biochem. Funct. 24, 483–489. 10.1002/cbf.1321 16397908

[B21] Karbownik-Lewińska M. Kokoszko-Bilska A. (2012). Oxidative Damage to Macromolecules in the Thyroid - Experimental Evidence. Thyroid. Res. 5, 25. 10.1186/1756-6614-5-25 23270549 PMC3542017

[B23] Kochman J. Jakubczyk K. Bargiel P. Janda-Milczarek K. (2021). The Influence of Oxidative Stress on Thyroid Diseases. Antioxidants 10, 1442. 10.3390/antiox10091442 34573074 PMC8465820

[B24] Kokoszko-Bilska A. Stepniak J. Lewinski A. Karbownik-Lewinska M. (2014). Protective Antioxidative Effects of Caffeic Acid Phenethyl Ester (CAPE) in the Thyroid and the Liver Are Similar to Those Caused by Melatonin. Thyroid. Res. 7, 5. 10.1186/1756-6614-7-5 25009581 PMC4090180

[B25] Koppenol W. H. Hider R. H. (2019). Iron and Redox Cycling. Do's and Don'ts. Free Radic. Biol. Med. 133, 3–10. 10.1016/j.freeradbiomed.2018.09.022 30236787

[B26] Lane D. J. R. Merlot A. M. Huang M. L.-H. Bae D.-H. Jansson P. J. Sahni S. (2015). Cellular Iron Uptake, Trafficking and Metabolism: Key Molecules and Mechanisms and Their Roles in Disease. Biochimica Biophysica Acta (BBA) - Mol. Cell. Res. 1853, 1130–1144. 10.1016/j.bbamcr.2015.01.021 25661197

[B27] Lewinski A. Karbownik M. (2002). REVIEW. Melatonin and the Thyroid Gland. Neuro. Endocrinol. Lett. 23 Suppl 1, 73–78. 12019356

[B28] Lipinski B. (2011). Hydroxyl Radical and its Scavengers in Health and Disease. Oxid. Med. Cell. Longev. 2011, 809696. 10.1155/2011/809696 21904647 PMC3166784

[B29] McCarthy P. L. Paterno G. D. Gillespie L. L. (2013). Protein Expression Pattern of Human MIER1 Alpha, a Novel Estrogen Receptor Binding Protein. J. Mol. Hist. 44, 469–479. 10.1007/s10735-012-9478-z PMC3921147 23277184

[B30] Mogulkoc R. Baltaci A. K. Oztekin E. Aydin L. Sivrikaya A. (2006). Melatonin Prevents Oxidant Damage in Various Tissues of Rats with Hyperthyroidism. Life Sci. 79, 311–315. 10.1016/j.lfs.2006.01.009 16464477

[B31] Nakanishi T. Kuragano T. Nanami M. Nagasawa Y. Hasuike Y. (2019). Misdistribution of Iron and Oxidative Stress in Chronic Kidney Disease. Free Radic. Biol. Med. 133, 248–253. 10.1016/j.freeradbiomed.2018.06.025 29958932

[B32] Osuna C. Reiter R. J. García J. J. Karbownik M. Tan D. X. Calvo J. R. (2002). Inhibitory Effect of Melatonin on Homocysteine-Induced Lipid Peroxidation in Rat Brain Homogenates. Pharmacol. Toxicol. 90, 32–37. 10.1034/j.1600-0773.2002.900107.x 12005111

[B33] Pachucki J. Wang D. Christophe D. Miot F. (2004). Structural and Functional Characterization of the Two Human ThOX/Duox Genes and Their 5′-flanking Regions. Mol. Cell. Endocrinol. 214, 53–62. 10.1016/j.mce.2003.11.026 15062544

[B34] Reiter R. Rosales-Corral S. Tan D.-X. Acuna-Castroviejo D. Qin L. Yang S.-F. (2017). Melatonin, a Full Service Anti-cancer Agent: Inhibition of Initiation, Progression and Metastasis. Ijms 18, 843. 10.3390/ijms18040843 PMC5412427 28420185

[B35] Rynkowska A. Stępniak J. Karbownik-Lewińska M. (2020). Fenton Reaction-Induced Oxidative Damage to Membrane Lipids and Protective Effects of 17β-Estradiol in Porcine Ovary and Thyroid Homogenates. Ijerph 17, 6841. 10.3390/ijerph17186841 PMC7559139 32962175

[B36] Rynkowska A. Stępniak J. Karbownik-Lewińska M. (2021). Melatonin and Indole-3-Propionic Acid Reduce Oxidative Damage to Membrane Lipids Induced by High Iron Concentrations in Porcine Skin. Membranes 11, 571. 10.3390/membranes11080571 34436334 PMC8400501

[B37] Schweizer U. Chiu J. Köhrle J. (2008). Peroxides and Peroxide-Degrading Enzymes in the Thyroid. Antioxidants Redox Signal. 10, 1577–1592. 10.1089/ars.2008.2054 18498223

[B38] Song Y. Driessens N. Costa M. De Deken X. Detours V. Corvilain B. (2007). Roles of Hydrogen Peroxide in Thyroid Physiology and Disease. J. Clin. Endocrinol. Metabolism 92, 3764–3773. 10.1210/jc.2007-0660 17666482

[B39] Stępniak J. Lewiński A. Karbownik-Lewińska M. (2013). Membrane Lipids and Nuclear DNA Are Differently Susceptive to Fenton Reaction Substrates in Porcine Thyroid. Toxicol. Vitro. 27, 71–78. 10.1016/j.tiv.2012.09.010 23022769

[B40] Stepniak J. Lewinski A. Karbownik-Lewinska M. (2021). Oxidative Damage to Membrane Lipids in the Thyroid - No Differences between Sexes. Drug Chem. Toxicol. 44, 655–660. 10.1080/01480545.2019.1643878 31373249

[B41] Stepniak J. Lewinski A. Karbownik-Lewinska M. (2018). Sexual Dimorphism of NADPH Oxidase/H2O2 System in Rat Thyroid Cells; Effect of Exogenous 17β-Estradiol. Ijms 19, 4063. 10.3390/ijms19124063 PMC6321217 30558263

[B42] Szanto I. Pusztaszeri M. Mavromati M. (2019). H2O2 Metabolism in Normal Thyroid Cells and in Thyroid Tumorigenesis: Focus on NADPH Oxidases. Antioxidants 8, 126. 10.3390/antiox8050126 PMC6563055 31083324

[B43] Valko M. Leibfritz D. Moncol J. Cronin M. T. D. Mazur M. Telser J. (2007). Free Radicals and Antioxidants in Normal Physiological Functions and Human Disease. Int. J. Biochem. Cell. Biol. 39, 44–84. 10.1016/j.biocel.2006.07.001 16978905

[B44] Xie J. Li Z. Tang Y. (2018). Successful Management of Multiple-Systemic Langerhans Cell Histiocytosis Involving Endocrine Organs in an Adult. Med. Baltim. 97, e11215. 10.1097/md.0000000000011215 PMC6039600 29952977

[B45] Zasada K. Karbownik-Lewinska M. (2015). Comparison of Potential Protective Effects of Melatonin and Propylthiouracil against Lipid Peroxidation Caused by Nitrobenzene in the Thyroid Gland. Toxicol. Ind. Health. 31, 1195–1201. 10.1177/0748233713491799 23723263

[B46] Ziech D. Franco R. Pappa A. Panayiotidis M. I. (2011). Reactive Oxygen Species (ROS)--induced Genetic and Epigenetic Alterations in Human Carcinogenesis. Mutat. Research/Fundamental Mol. Mech. Mutagen. 711, 167–173. 10.1016/j.mrfmmm.2011.02.015 21419141

[B47] Zou D.-M. Sun W.-L. (2017). Relationship between Hepatitis C Virus Infection and Iron Overload. Chin. Med. J. Engl. 130, 866–871. 10.4103/0366-6999.202737 28345552 PMC5381322

